# High expression of Bruton’s tyrosine kinase (BTK) is required for EGFR-induced NF-κB activation and predicts poor prognosis in human glioma

**DOI:** 10.1186/s13046-017-0600-7

**Published:** 2017-09-25

**Authors:** Chenglong Yue, Mingshan Niu, Qian Qian Shan, Ting Zhou, Yiming Tu, Peng Xie, Lei Hua, Rutong Yu, Xuejiao Liu

**Affiliations:** 1 Insititute of Nervous System Diseases, Xuzhou Medical University, Xuzhou, Jiangsu China; 2 Brain Hospital, the Affiliated Hospital of Xuzhou Medical University, Xuzhou, Jiangsu China; 3 Blood Diseases Institute, Jiangsu Key Laboratory of Bone Marrow Stem Cell, Xuzhou Medical University, Xuzhou, Jiangsu China; 4 Department of Gastroenterology, Xuzhou Medical University, Xuzhou, Jiangsu China; 5 Department of Neurosurgery, Huai’an Hospital Affiliated of Xuzhou Medical University and Huai’an Second People’s Hospital, Huaian, Jiangsu China

**Keywords:** Glioma, BTK, Ibrutinib, Clinical outcome, NF-κB pathway

## Abstract

**Background:**

Malignant glioma is the most common primary brain tumor in adults and has a poor prognosis. However, there are no effective targeted therapies for glioma patients. Thus, the development of novel targeted therapeutics for glioma is urgently needed.

**Methods:**

In this study, we examined the prognostic significance BTK expression in patients with glioma. Furthermore, we investigated the mechanism and therapeutic potential of ibrutinib in the treatment of human glioma in vitro and in vivo.

**Results:**

Our data demonstrate that high expression of BTK is a novel prognostic marker for poor survival in patients with glioma. BTK-specific inhibitor ibrutinib effectively inhibits the proliferation, migration and invasion ability of glioma cells. Furthermore, ibrutinib can induce G1 cell-cycle arrest by regulating multiple cell cycle-associated proteins. More importantly, we found that BTK inhibition significantly blocks the degradation of IκBα and prevents the nuclear accumulation of NF-κB p65 subunit induced by EGF in glioma cells.

**Conclusions:**

Taken together, our study suggests that BTK is a novel prognostic marker and molecular therapeutic target for glioma. BTK is required for EGFR-induced NF-κB activation in glioma cells. These findings provide the basis for future clinical studies of ibrutinib for the treatment of glioma.

## Background

Glioma is the most common form of malignant brain cancer in humans. Glioblastoma multiforme (GBM) is the most common and lethal subtypes of glioma [1, 2]. Despite aggressive treatment with surgery, radiation and chemotherapy, the median survival for patients with GBM is merely 12 to 15 months [3, 4]. Thus, understanding the molecular mechanism involved in the oncogenesis of glioma is essential for developing more effective treatment and improving patient survival.

NF-κB is involved in a myriad of activities related to cellular functions [5]. The NF-κB pathway plays an important role in promoting tumor pathobiology and confers a negative prognosis in patients with glioma [6, 7]. In glioma cells, constitutive activation of NF-κB has been shown to promote tumor cell growth and survival [8, 9]. Inhibition of NF-κB activity can induce apoptosis of glioma cells. A large number of preclinical studies suggested that the NF-κB signaling network is a promising target for glioma treatment [10]. However, the precise role of NF-κB activation in the pathogenesis of glioma has not been fully characterized.

The Bruton’s tyrosine kinase (BTK) belongs to the cytoplasmic tyrosine kinase family and is important for B-cell receptor signaling [11, 12]. Loss of BTK results in defective BCR-induced activation of both canonical and noncanonical NF-κB pathways. Ibrutinib is a selective covalent inhibitor of BTK and has excellent clinical activity in patients with chronic lymphocytic leukemia and lymphoma. More recently, ibrutinib has been shown to suppress the growth of gastric, breast and colon tumors in mouse model [13, 14].

As NF-κB pathway is essential for glioma cell proliferation and survival, we therefore investigated whether ibrutinib is involved in NF-κB activation in glioma cells. In the present study, we examined the prognostic significance BTK expression in patients with glioma. Furthermore, we investigated the mechanism and therapeutic potential of ibrutinib in the treatment of human glioma. Our finding provides the basis for further clinical investigations of ibrutinib in human glioma.

## Methods

### Tissue samples

The human glioma specimens and non-tumorous brain tissues were collected from the Department of Neurosurgery of Xuzhou Medical University. This study was approved by the Ethics Committee of the Affiliated Hospital of Xuzhou Medical University.

### Cell lines and reagents

U87 EGFRvIII, U87, U251, HA1800 cells were maintained in DMEM medium supplemented with 10% fetal bovine serum at 37 °C with 5% CO_2_. Ibrutinib was purchased from MedChem Express. EGF was purchased from RD systems. Antibodies against p65, Bcl-2, Bax, IκBα, actin, cyclin D1, p27, p21, BTK were obtained from Cell Signaling Technology (CST, Beverly, MA, USA).

### Cell viability assay

Cell viability was evaluated by a Cell Counting Kit-8 (Japanese colleagues) assay following the manufacturer’s instructions [15]. U87 and U251 cells were harvested and seeded in 96-well plates. After 24 h incubation, the cells were treated with different concentrations of ibrutinib and cultured for 72 h. Next, CCK8 was added to each well and incubated for 2 h. The absorbance was measured at 450 nm using the microplate reader.

### EdU assay

Cell proliferation was assessed by 5-ethynyl-2′-deoxyuridine (EdU) fluorescence staining using the Cell-Light™ EdU DNA Cell Proliferation Kit (Ruibo Biotech, Guangzhou, China). The glioma cells were seeded in 96-well culture plates and incubated overnight. Subsequently, the cells were treated with ibrutinib at various concentrations for 12 h and incubated with 50 μL EdU for 4 h at 37 °C. The cells were then fixed with 4% paraformaldehyde for 15 min and treated with 0.5% Triton X-100 for 20 min. Subsequently, the cells were incubated with 100 μL of 1× Apollo® reaction cocktail for 30 min and stained with DAPI for 15 min. After washing with PBS, the cells were examined with fluorescence microscopy and photographed (Olympus, Japan).

### Cell cycle assay

For the cell cycle analysis [16], U87 and U251 cells were treated with or without ibrutinib for 24 h. The cells were collected and fixed in 70% ethanol. After washing with PBS, the cells were stained with PI solution that contained 25 μg/mL Rnase and 50 μg/mL PI for 30 min. Subsequently, the cells were assayed on a FACSCalibur (Becton-Dickinson) and analyzed by CellQuest Pro software (Becton-Dickinson).

### Wound healing assay

U87 and U251 cells were seeded in 6-well plates and allowed to attach overnight. A scratch was made in the middle of the well with a plastic pipette tip. The cells were incubated in serum-free media and treated with either 0.1% DMSO or ibrutinib. After incubation, five randomly selected fields at the lesion border were acquired under microscope (Olympus IX71).

### Transwell invasion assay

Cell invasion assay was performed using a transwell system. Briefly, transwell was coated with 50 μL of matrigel in cold serum-free medium at a final concentration of 1 mg/mL. U87 and U251 cells were treated with either 0.1% DMSO or ibrutinib in serum-free media and added to the top chamber. In the lower chamber, the DMEM media containing 10% FBS was added. After 30 h of incubation, the cells were stained with 0.1% crystal violet for 5 min. The observation of the migratory cells was recorded with microscopy.

### Western blot analysis

Cells were plated in six-well plates and incubated with various concentrations of ibrutinib. The whole, cytoplasmic or nuclear cell extracts of treated cells were extracted and evaluated using 10% SDS-polyacrylamide gels electrophoresis. After electrophoresis, the proteins were transferred to PVDF membranes. Membranes were subsequently blocked with 5% defatted milk powder for 1 h at room temperature and incubated with indicated antibodies. The signals were detected using the enhanced chemiluminescence detection system.

### In vivo glioma model

Fifteen BALB/c nude mice were obtained from the experimental animal center of Xuzhou Medical University. The mice were transplanted with U87 cells at 6 weeks old. The U87 cells were subcutaneously injected into mice at a dose of 1 × 10^6^ cells/mouse. The mice were randomly divided into the following three treatment groups (*n* = 5 per group): 50 mg/kg, 100 mg/kg, and vehicle. The drug and vehicle were intraperitoneally injected every day. Tumor growth was monitored periodically to minimize the difference in tumor size between groups. After 16 days of injection, mice were sacrificed and prepared for immunofluorescence analysis.

U87 cells (3 × 10^5^ cells per mouse) were intracranially injected into the right striatum of nude mice using a small animal stereotactic apparatus. After 7 days of tumor injection, the tumor-bearing mice were randomly divided into one of the following three treatment groups: ibrutinib at 50 mg/kg, ibrutinib at 100 mg/kg and vehicle. The drugs and vehicle were delivered daily via intraperitoneal injections.

### Immunofluorescence analysis

The removed tumors were fixed in 4% paraformaldehyde overnight. The tumor sections were incubated with 0.3% TritonX-100 and 10% goat serum. Subsequently, the cells were incubated with primary antibody and Alexa-594-conjugated secondary antibody for 1 h, respectively. The cellular DNA was stained with DAPI for 15 min. All sections were examined and photographed with a microscope with an attached fluorescence detector (IX71 Olympus).

### BTK expression and survival analysis in patients with glioma

BTK gene expression datasets were obtained from R2: microarray analysis and visualization platform (http://r2.amc.nl). The Kaplan-Meier analysis was conducted online, and cutoff values for separating high and low expression groups were determine by auto scan. The Kaplan scanner was used to determine the best cut-off values for the level of gene expression.

### Statistical analysis

The results are presented as the means ± SEM of three independent experiments. Comparisons of the mean values between the control and treated groups were performed using Student’s *t* test. A Kaplan-Meier survival curve was used for the survival analysis. *P* < 0.05 is considered statistically significant.

## Results

### High BTK expression predicts poor outcome in patients with glioma

**Fig. 1:**
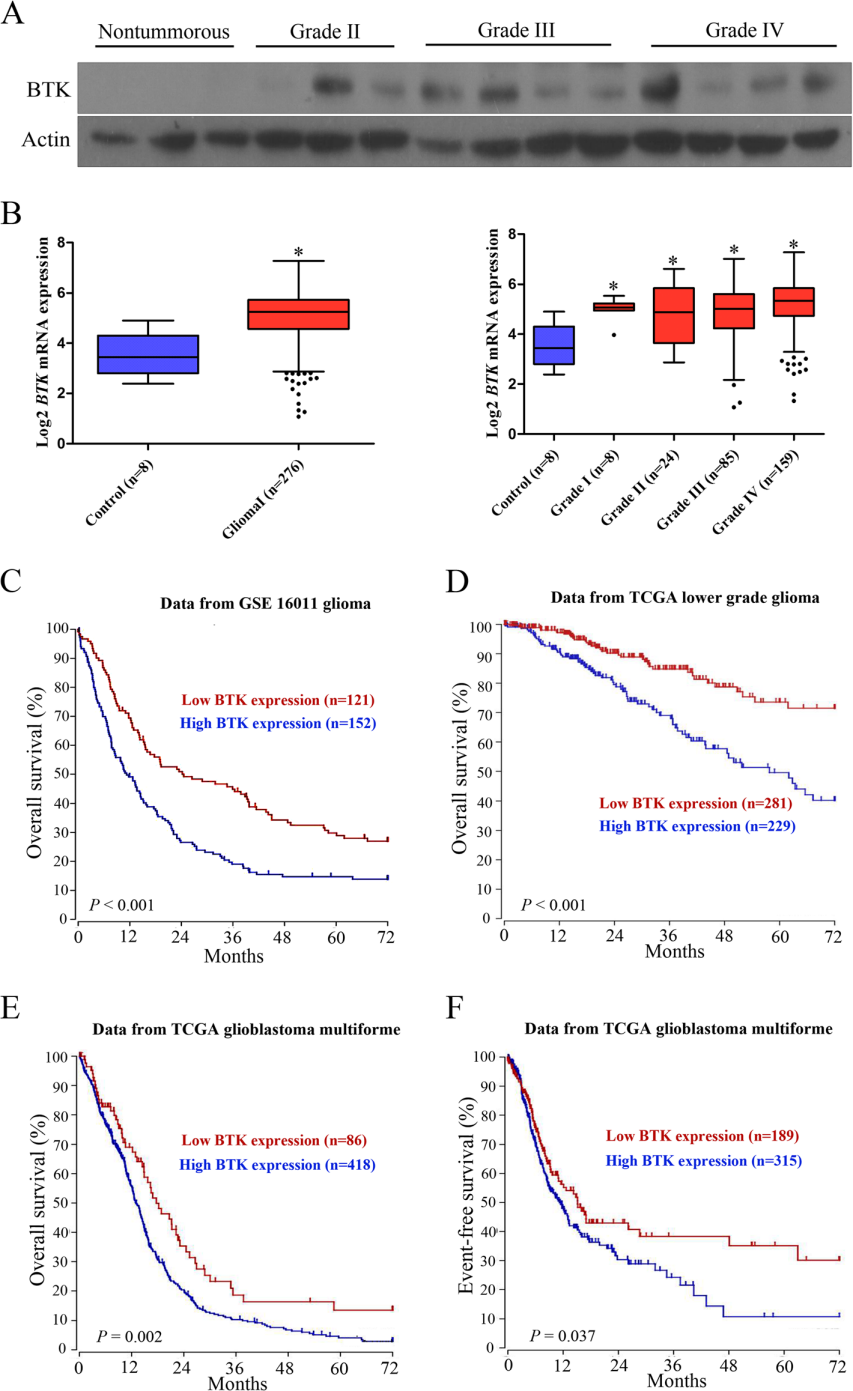
High expression of BTK correlates with poor outcome in glioma patients**.** (**a**) Total protein extracts isolated from non-tumorous brain tissues and glioma tissues were evaluated through western blotting analysis. (**b**) The mRNA expression of BTK was high in glioma patients. Microarray gene expression data were obtained from GEO database (accession number GSE16011). (**c**) Kaplan-Meier survival analysis of glioma patients was performed using GEO dataset (accession number GSE16011). BTK was high expression in 152 out of 273 glioma cases. (**d**) Overall survival analysis of lower grade glioma (LGG) patients was performed using TCGA LGG dataset. (**e**) Overall survival analysis of GBM patients was performed using TCGA GBM dataset. (**f**) Event-free survival analysis of GBM patients was performed using TCGA GBM dataset

To investigate the protein expression profile of BTK in gliomas, Western blot analysis was used in glioma specimens and normal brain tissues. As shown in Fig. 1A, BTK was highly expressed in glioma samples compared with non-tumorous brain tissues. We then analyzed the mRNA expression of BTK in human normal brain and glioma samples using GEO microarray dataset (GSE16011). As shown in Fig. 1B, BTK expression was significantly higher in glioma than normal samples. However, we failed to observe any significant differences between the grades of glioma. Next, we examined the correlation of BTK gene expression with patient outcome using microarray dataset. As shown in Fig. 1C, the glioma patients expressing high levels of BTK showed statistically poor outcome compared with the low expression group (GSE16011 dataset). We also found that high BTK expression levels were associated with poor prognosis in patients with lower grade glioma using TCGA LGG dataset (Fig. 1D). Furthermore, high BTK expression was associated with poor outcome in patients with GBM, as the overall and event-free survival were both markedly reduced in cases exhibiting high BTK expression (Fig. 1E and F). These results suggest that high expression of BTK is a poor prognostic marker for glioma patients.

### Ibrutinib inhibits proliferation of glioma cells.

**Fig. 2:**
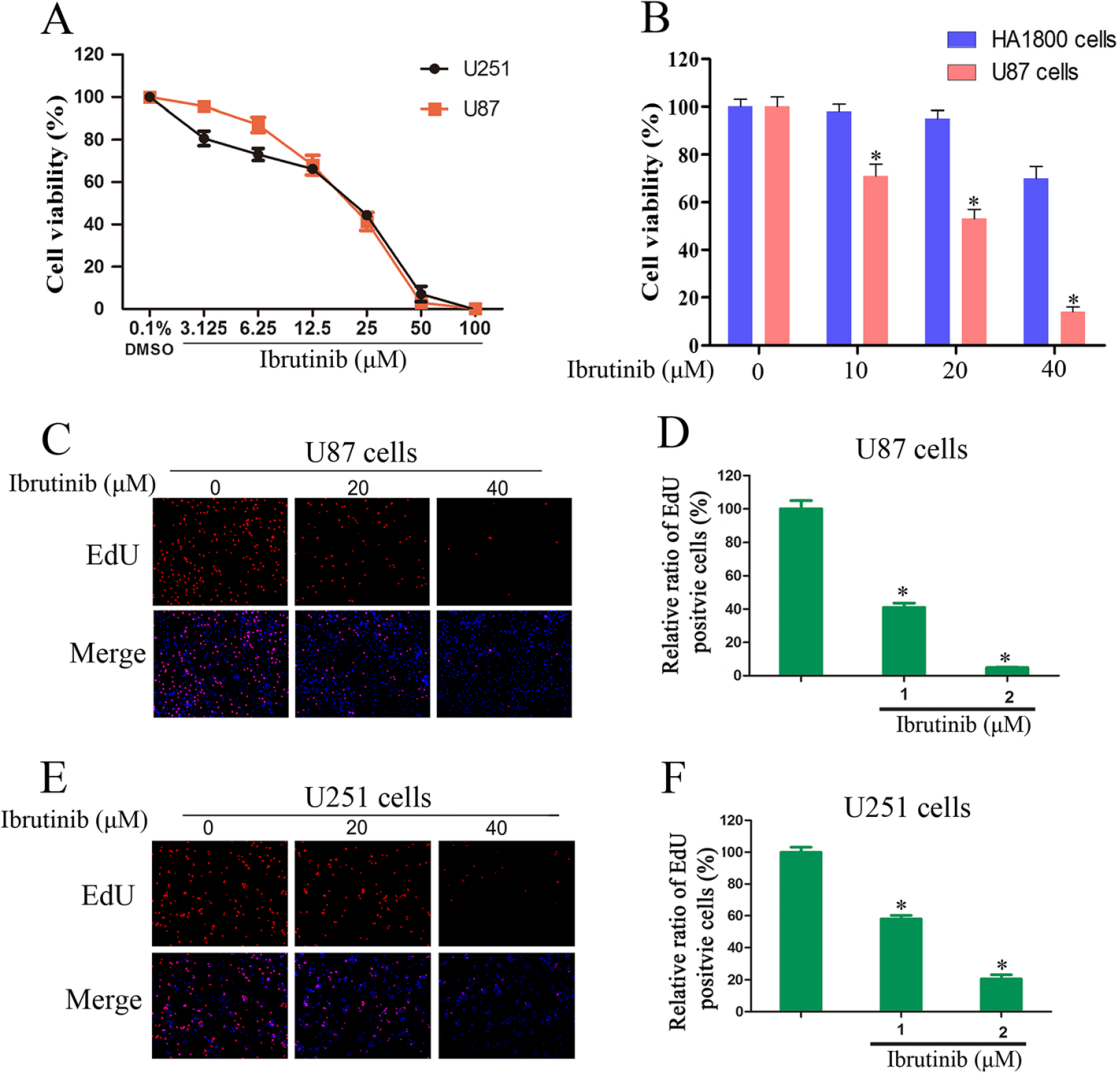
BTK inhibitor ibrutinib suppresses the proliferation of glioma cells. (**a**) U87 and U251 cells were treated with the indicated concentration of ibrutinib for 72 h. The cell viability was measured using CCK-8 assays. (**b**) HA1800 and U87 cells were treated with the indicated concentration of ibrutinib for 72 h. The cell viability was measured using CCK-8 assays. (**c-f**) The Ibrutinib-induced inhibition of DNA synthesis was determined by EdU incorporation assays. The EdU incorporation rate was presented as the ratio of EdU positive cells to total DAPI positive cells

To examine the effect of ibrutinib on the growth of glioma, we evaluated the viability of glioma cells treated with ibrutinib using the CCK-8 assay. As shown in Fig. 2A, ibrutinib significantly inhibited cell growth of U251 and U87 cells in a dose dependent manner. The sensitivity to ibrutinib was similar between U87 and U251 cells. Furthermore, the reduced viability was much more pronounced in glioma cells, while normal human astrocyte cell viability was only slightly impaired at the high concentration (Fig. 2B). To confirm the efficacy of ibrutinib in glioma cells, we tested the cell proliferation using EdU assay. Our results demonstrated that ibrutinib treatment resulted in a significant reduction of EdU-positive cells compared with the control group (Fig. 2C-2F). In conclusion, these data suggest that ibrutinib can effectively inhibit the proliferation of glioma cells.

### Ibrutinib inhibits the migration and invasion of glioma cells

**Fig. 3:**
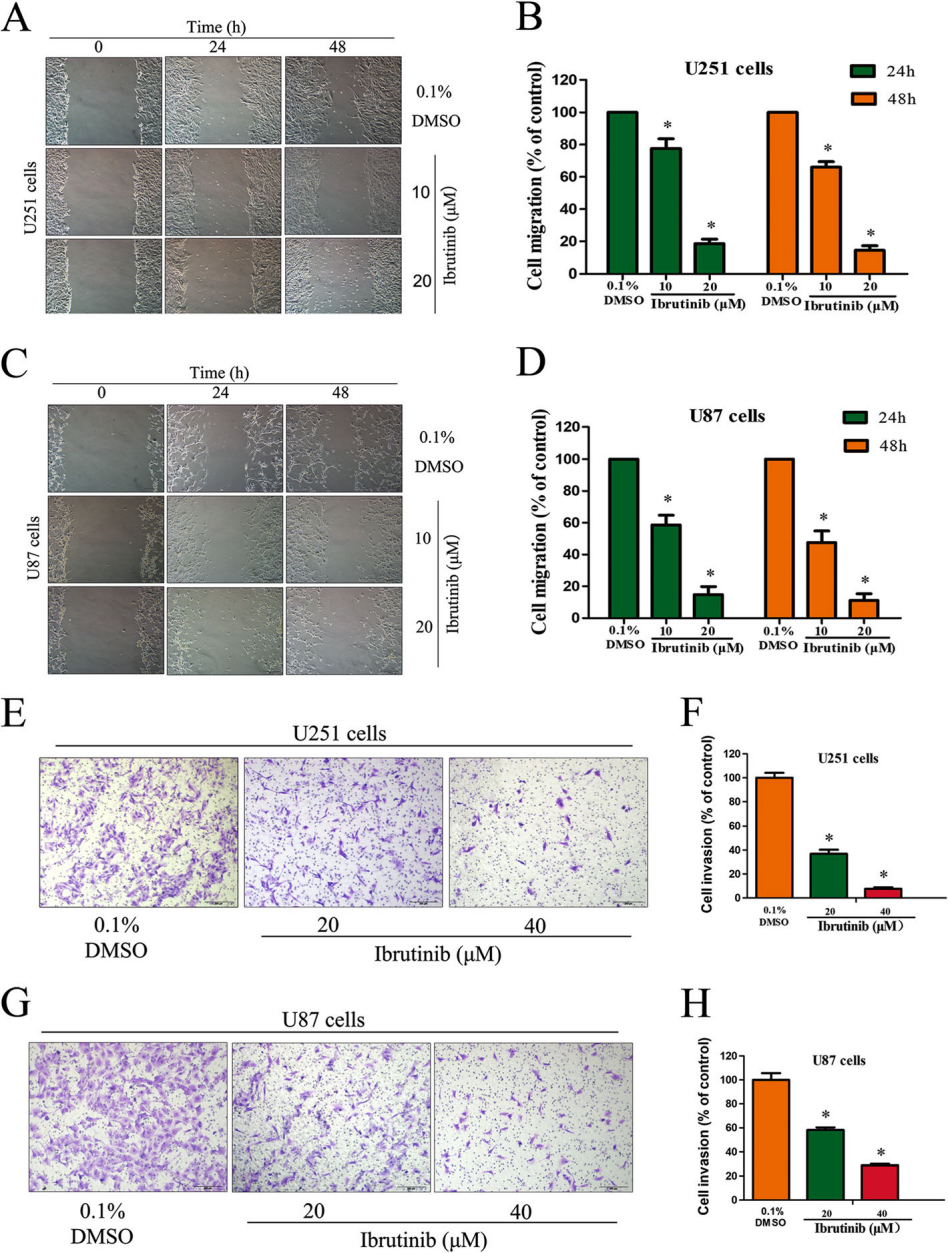
Ibrutinib inhibits the migration and invasion of glioma cells. (**a**-**d**) Ibrutinib reduced the migration ability of U87 and U251 cells as examined by wound healing assay. (**e**-**h**) Ibrutinib reduced the invasion ability of U87 and U251 cells as examined by transwell assay. The positive numbers of cells were counted and normalized to the control group. The data are expressed as the mean ± SEM from three independent experiments

To determine the effects of ibrutinib on migration and invasion of glioma cells, we performed the in vitro wound healing and transwell invasion assays. We observed that ibrutinib treatment inhibited migration of U87 and U251 cells in a dose dependent manner (Fig. 3A-3D). Compared with control groups, the migratory areas of U87 cells were reduced to 58.6% and 14.8% in response treatment with 20 and 40 μM ibrutinib, respectively. As shown in Fig. 3E-3F, ibrutinib treatment led to a significant decrease in the invasion activity of glioma cells in the transwell assay. The exposure of U87 cells to 20 and 40 μM ibrutinib reduced invasion rate of these cells to 58.2% and 28.9%, respectively. Taken together, our data demonstrate that ibrutinib can effectively inhibit the migration and invasion ability of glioma cells.

### Ibrutinib induces G1 arrest through regulating the expression of cell cycle-associated proteins

**Fig. 4:**
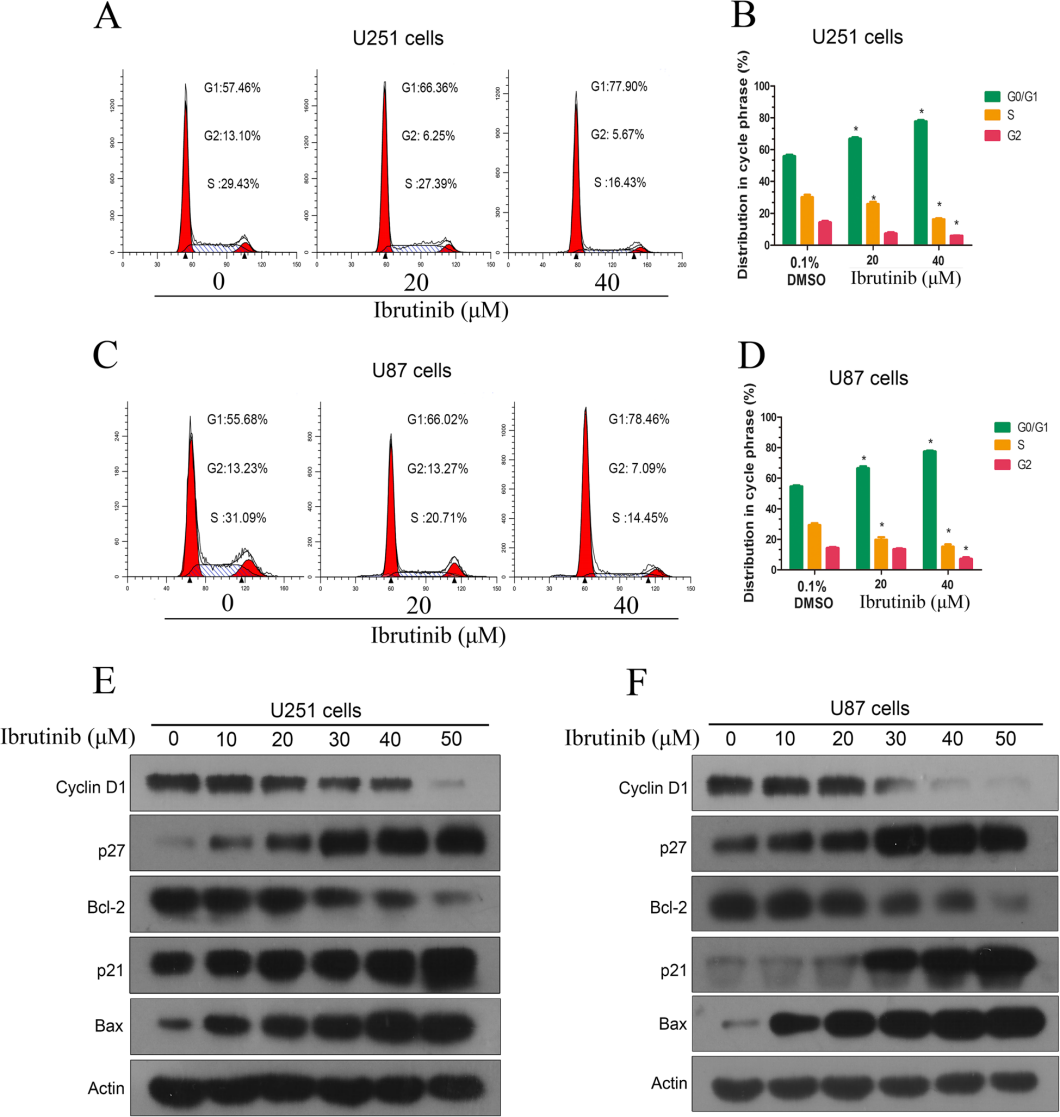
Ibrutinib induces G1 phase arrest through regulating the expression of cell cycle-related proteins. (**a**-**d**) U251 and U87 cells were incubated with ibrutinib for 12 h. The cell cycle profile was evaluated using flow cytometry. The data from three independent experiments are expressed as the mean ± SEM. (**e** and **f**) The effects of ibrutinib on the expression levels of cell cycle-related protein. U87 and U251 cells were treated with ibrutinib for 12 h. The protein extracts were examined using Western blot analysis with the indicated antibodies

To determine the mechanism underlying the anti-proliferative activity of ibrutinib, flow cytometry assay was performed. We found that ibrutinib treatment induced a significant increase in G1 phase followed the increasing concentrations both in U87 and U251 cells (Fig. 4A-4D). The exposure of U87 cells to 20 and 40 μM ibrutinib increased the percentage of cells in the G1 phase from 55.7% to 66.1% and 78.5%, respectively. Similar results were detected in U251 cells in response to ibrutinib treatment. These data indicate that ibrutinib inhibits proliferation of glioma cells through inducing G1 cell-cycle arrest.

To further explore the mechanism by which ibrutinib regulates cell cycle, we examined the expression levels of several regulators of cell cycle progression. As shown in Fig. 4E and F, the levels of the cell cycle inhibitory proteins p21 and p27 were increased in ibrutinib-treated glioma cells. However, ibrutinib treatment significantly decreased the expression levels of cyclin D1 in a dose dependent manner. Our data also showed that the expression of Bcl-2 (anti-apoptotic protein) was noticeably decreased in response to the treatment with ibrutinib, while the expression of Bax (pro-apoptotic protein) protein was steadily increased. These data confirm that ibrutinib induces G1 cell-cycle arrest by regulating multiple cell cycle- and apoptosis-associated proteins.

### Ibrutinib suppresses glioma xenograft tumorigenesis in vivo

**Fig. 5:**
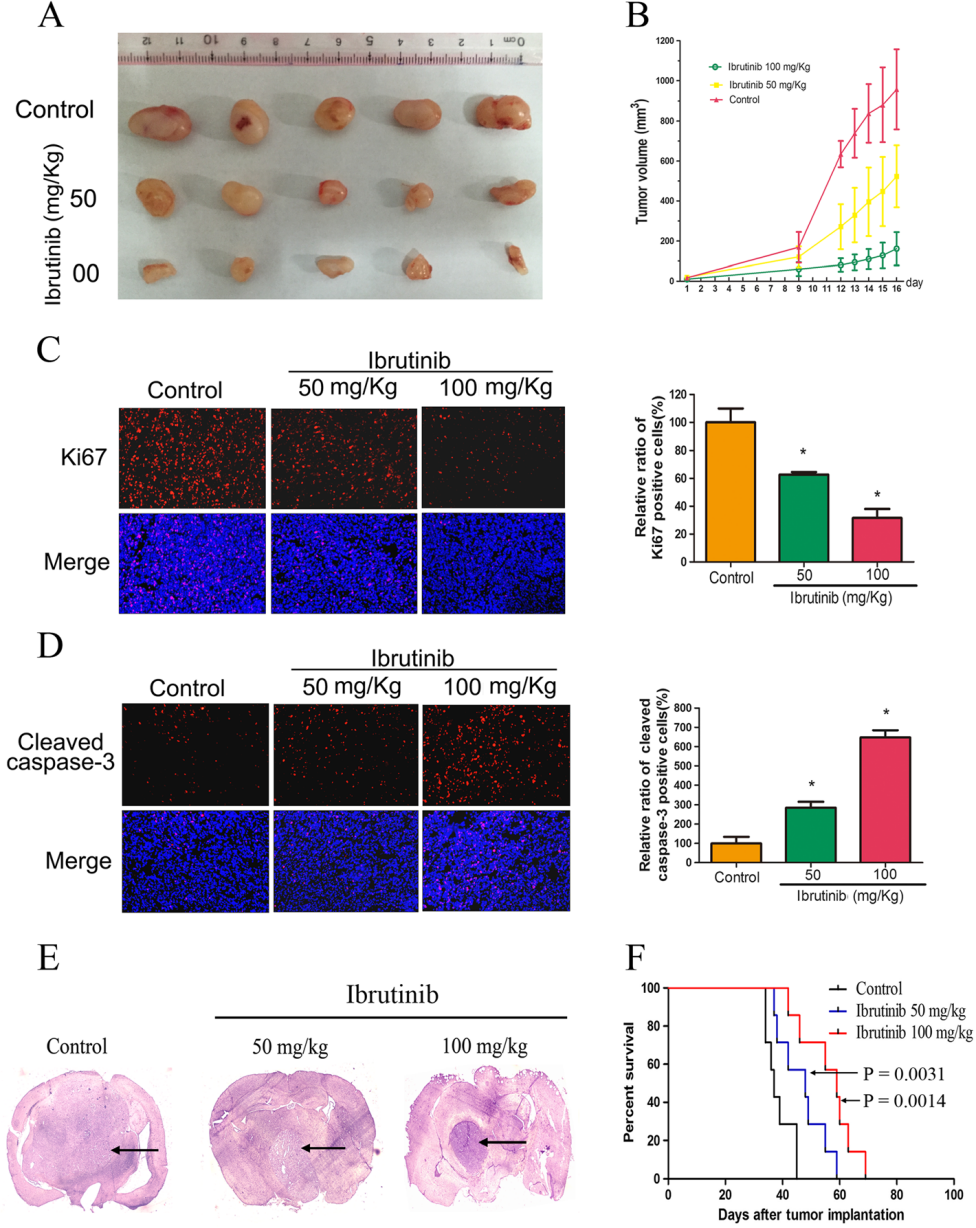
The anti-tumor effect of ibrutinib in vivo. (**a**) U87 cells (1× 10^6^ cells per mouse) were subcutaneously injected into nude mice. The mice were treated with ibrutinib daily for 15 days. Subsequently, the mice were sacrificed and tumors were collected. Representative tumors isolated from the control and ibrutinib treated groups. (**b**) After ibrutinib treatment, the mean tumor volumes were assessed at the indicated days. (**c**-**d**) The Ki-67 and cleaved caspase-3 immunohistochemistry staining of U87 xenografts. The positive numbers of cells were counted and normalized to the control group. These data are presented as the means ± SEM of three independent experiments. (**e**) Representative images of H&E staining of coronal sections from mouse brains with orthotopic tumors. (**f**) The survival of mice with tumors derived from the vehicle or ibrutinib-treated groups was measured by Kaplan-Meier survival curves

To investigate the effects of ibrutinib on glioma growth in vivo, we established a subcutaneous exnograft tumor model of glioma cells. We observed that the transplanted U87 cells in ibrutinib-treated mice were visibly smaller than those in the vehicle-treatment mice (Fig. 5A). As shown in Fig. 5B, ibrutinib treatment suppressed proliferation of U87 cells in vivo as early as 9 day post-treatment. On day 16 after ibrutinib treatment, the mean tumor volumes were 523 and 161 mm^3^ in response to 50 and 100 mg/Kg ibrutinib treatment, respectively. However, the mean tumor volume of the vehicle group was 957 mm^3^. These data demonstrate that ibrutinib significantly suppresses proliferation of U87 cells in vivo.

To further evaluate the effect of ibrutinib on glioma cell growth in vivo, we examined the marker for cell proliferation by performing immunohistochemistry on tumor tissues isolated from ibrutinib-treated mice. The Ki67 and cleaved caspase-3 were used to show cell proliferation and apoptosis in xenografts, respectively. Compared to the control group, the percentages of Ki67 positive cells were significantly decreased to 62.7% and 31.6% in 50 and 100 mg/Kg ibrutinib-treated groups, respectively (Fig. 5C). In contrast, the percentage of cleaved caspase-3 positive tumor cells was significantly increased in the ibrutinib-treated group (Fig. 5D). These data suggest that therapeutic targeting of BTK using the ibrutinib has powerful suppressive effects on human glioma cells in vivo. In an intracranial xenograft model, the transplanted gliomas in ibritinib-treated mice were visibly smaller than those in the vehicle-treated mice (Fig. 5E). Consequently, the ibritinib-treated mice exhibited significantly increased survival (Fig. 5F).

### BTK is crucial for EGF-induced NF-κB activation in glioma cells

**Fig. 6:**
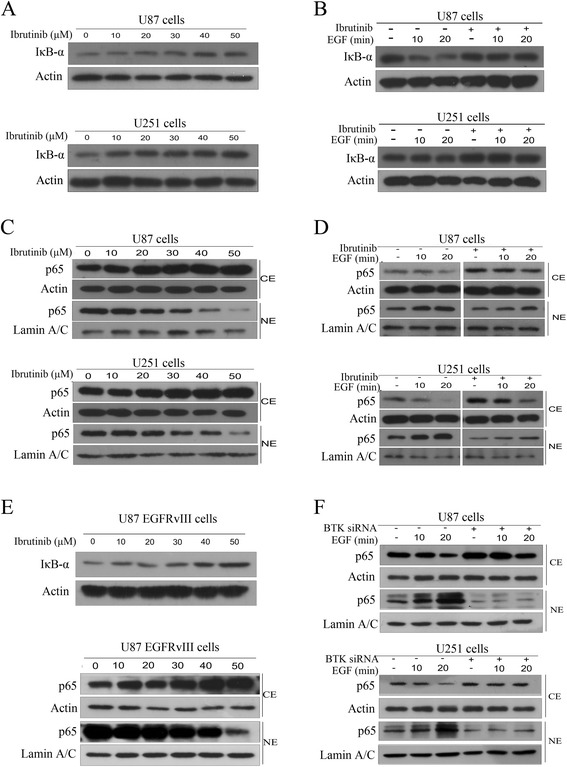
Ibrutinib inhibits EGFR-induced NF-κB activation in glioma cells. (**a**) The cells were treated with indicated concentrations of ibrutinib for 3 h. The whole cell extracts were analyzed by Western blot analysis using IκB-α antibody. (**b**) Ibrutinib suppressed EGF-induced degradation of IκB-α. U87 and U251 cells were pretreated with or without ibrutinib (30 μM) for 2 h. The cells were then stimulated with EGF (100 ng/mL) for the indicated times. The expression of IκB-α was analyzed by Western blot analysis. (**c**) Ibrutinib inhibited nuclear translocation of p65 in glioma cells. The cells were treated with indicated concentrations of ibrutinib for 3 h. The nuclear extracts (NE) and cytoplasmic extracts (CE) were analyzed by Western blotting. (**d**) Ibrutinib inhibited EGF-induced nuclear translocation of p65 in glioma cells. U87 and U251 cells were pretreated with or without ibrutinib (30 μM) for 2 h. The cells were then stimulated with EGF (100 ng/mL) for the indicated times. The cytoplasmic and nuclear protein extracts were analyzed by Western blotting. (**e**) Ibrutinib inhibited degradation of IκB-α and nuclear translocation of p65 in U87 EGFRvIII cells. (**f**) BTK knockdown cells were stimulated with EGF (100 ng/mL) for indicated time and then cell lysates were subjected to immunoblotting analysis using indicated antibodies

To gain further insight into the mechanism of ibrutinib-induced cytotoxicity, we evaluated the effects of ibrutinib on NF-κB pathway. As shown in Fig. 6A, ibrutinib treatment significantly increased the expression of IκBα in a dose-dependent manner. Furthermore, exposure of glioma cells to ibrutinib resulted in a decreased nuclear accumulation of NF-κB p65 subunit compared with control (Fig. 6C). We then asked whether BTK was required for EGFR-induced NF-κB activation. As shown in Fig. 6B, EGF could effectively induce degradation of IκBα in glioma cells, but this degradation was completely abolished by ibrutinib pretreatment. Consistent with above results, we found that EGF stimulation induced a significant increase in nuclear p65 level and a decrease in cytoplasmic p65. However, BTK inhibition markedly prevented the EGF-induced nuclear accumulation of p65 in glioma cells (Fig. 6D). Furthermore, our data demonstrated that ibrutinib could inhibit the degradation of IκBα and nuclear translocation of p65 in U87 EGFRvIII cells (Fig. 6E). To further demonstrate the role of BTK in EGFR-induced NF-κB activation in glioma cells, U87 and U251 cells were transduced with BTK siRNA. We found that knockdown of BTK significantly suppressed EGFR-induced NF-κB activation in glioma cells (Fig. 6F). Taken together, our data demonstrate that BTK is required for EGFR-induced NF-κB activation in glioma cells.

## Discussion

Despite advances in clinical therapies, the prognosis for malignant glioma patients is very poor. Up to now, there are no effective targeted therapies for glioma patients [17, 18]. Thus, the development of novel targeted therapeutics for glioma is urgently needed. In this study, we found that high expression of BTK predicts poor outcome of patients with glioma. Furthermore, BTK inhibition suppresses the proliferation of glioma cells in vitro and in vivo, and abolishes the EGFR-induced NF-κB activation.

BTK is a non-receptor tyrosine kinase belonging to the Tec kinase family. High expression of BTK is associated with the tumorigenesis of leukemia and lymphoma [19, 20]. Recently, BTK has been shown to highly express in several solid tumors and may contribute to tumor development [14]. Our data demonstrate that high expression of BTK is a novel poor prognostic marker for patients with glioma. The expression of BTK is higher in glioma than normal samples. However, there are no significant differences between the four grades of glioma. Our data suggest that BTK is up-regulated in a group of glioma patients and may be an important regulator of this disease.

Ibrutinib is the first BTK-specific inhibitor and approved for the clinical treatment of mantle cell lymphoma and chronic lymphocytic leukemia [21, 22]. Ibrutinib has recently been shown to have an-tumor activity in some solid cancers [23]. More recently, BTK has been shown to also play a tumorigenic role in GBM and associate with its sphere-forming potential [24]. Consistent with the report, we found that ibrutinib can significantly inhibit the proliferation of glioma cells in vitro and in vivo through inducing G1 cell cycle arrest. Furthermore, ibrutinib treatment increases the expression levels of the cell cycle inhibitory proteins p21 and p27, and decreases the levels of Cyclin D1 in glioma cells. Ibrutinib inhibits glioma cell growth also through the regulation of Bcl-2/Bax and activation of caspase-3. Taken together, the anti-proliferative effect of ibrutinib in glioma cells was associated with cell cycle arrest and induction of apoptosis. Our results indicate that ibrutinib can be evaluated as novel therapeutic agent for glioma tumor. A recent study demonstrate that the combination of ibrutinib and temozolomide (TMZ) exert more inhibitory effect [24]. Thus, the combination of TMZ and ibrutinib may be a rational development of therapeutic approaches and clinical trials for glioma treatment.

The genetic alterations of EGFR pathway are the most common oncogenic drivers in glioma [25]. The hyperactivation of EGFR signaling is a primary contributor to glioma initiation and progression [26]. More importantly, EGFR promotes glioma cells survival and chemotherapy resistance through Akt-independent activation of NF-κB pathway [27]. EGFR signaling is an important driver of NF-κB activation in GBM [28]. However, the molecular mechanism of EGFR-induced activation of NF-κB in glioma is still unclear. In present study, we found that BTK inhibition can significantly block the degradation of IκBα and prevent the nuclear accumulation of NF-κB p65 subunit induced by EGF in glioma cells. IκBα is a major negative regulator of NF-κB that prevents nuclear translocation of p65 [29]. The nuclear accumulation of p65 can regulate the transcription of several genes involved in cell proliferation, survival and invasion of glioma [30]. Our results suggest that BTK is a novel critical mediator of EGFR-induced NF-κB activation in glioma cells.

## Conclusions

In summary, this is the first report that overexpression of BTK are correlated with poor outcome in almost 50% of glioma patients. Our results demonstrate that ibrutinib can significantly inhibit the proliferation of glioma cells in vitro and in vivo through inducing G1 phase arrest and modulating the expression of cell cycle regulators. More importantly, BTK inhibition effectively blocked both baseline and EGFR-induced activation of NF-κB pathway mediating glioma cell proliferation and survival. These findings provide the basis for future clinical studies of ibrutinib for the treatment of glioma.

## Abbreviations

BTK: Bruton’s tyrosine kinase
CCK-8: Cell counting kit-8
EdU: 5-ethynyl-2′-deoxyuridine
EGF: Epidermal growth factor
EGFR: Epidermal growth factor receptor
GBM: Glioblastoma multiforme
